# Brain structural changes and neuropsychological impairments in male polydipsic schizophrenia

**DOI:** 10.1186/1471-244X-12-210

**Published:** 2012-11-26

**Authors:** Tomohisa Nagashima, Makoto Inoue, Soichiro Kitamura, Kuniaki Kiuchi, Jun Kosaka, Koji Okada, Naoko Kishimoto, Toshiaki Taoka, Kimihiko Kichikawa, Toshifumi Kishimoto

**Affiliations:** 1 Department of Psychiatry, Nara Medical University, Kashihara, Nara, Japan; 2 National Hospital Organization Yamato Mental Medical Center, Yamatokoriyama, Nara, Japan; 3 Sakai City Mental Health Center, Sakai, Osaka, Japan; 4 Department of Radiology, Nara Medical University, Kashihara, Nara, Japan

**Keywords:** Schizophrenia, Polydipsia, Volumetry, MRI, Neuropsychological impairment, Brief assessment of cognition in schizophrenia

## Abstract

**Background:**

Polydipsia frequently occurs in schizophrenia patients. The excessive water loading in polydipsia occasionally induces a hyponatremic state and leads to water intoxication. Whether polydipsia in schizophrenic patients correlates with neuropsychological impairments or structural brain changes is not clear and remains controversial.

**Methods:**

Eight polydipsic schizophrenia patients, eight nonpolydipsic schizophrenia patients, and eight healthy controls were recruited. All subjects underwent magnetic resonance imaging (MRI) and neuropsychological testing. Structural abnormalities were analyzed using a voxel-based morphometry (VBM) approach, and patients’ neuropsychological function was assessed using the Brief Assessment of Cognition in Schizophrenia, Japanese version (BACS-J).

**Results:**

No significant differences were found between the two patient groups with respect to the clinical characteristics. Compared with healthy controls, polydipsic patients showed widespread brain volume reduction and neuropsychological impairment. Furthermore, the left insula was significantly reduced in polydipsic patients compared with nonpolydipsic patients. These nonpolydipsic patients performed intermediate to the other two groups in the neuropsychological function test.

**Conclusions:**

It is possible that polydipsia or the secondary hyponatremia might induce left insula volume reduction. Furthermore, this structural brain change may indirectly induce more severe neuropsychological impairments in polydipsic patients. Thus, we suggest that insula abnormalities might contribute to the pathophysiology of polydipsic patients.

## Background

Schizophrenia affects about 1% of the population worldwide [1], and induces neuropsychological impairment [2], as well as structural brain changes [3]. Among the many comorbidities associated with schizophrenia, polydipsia is defined as either chronic or intermittent ingestion of large volumes of water. Polydipsia occurs frequently among patients with schizophrenia and may be present in more than 20% of chronic psychiatric inpatients [4]. Polydipsia is not explained by medically induced polyuria, but it may lead to hyponatremic symptoms and can cause neurological symptoms. These neurological symptoms include: nausea, vomiting, delirium, ataxia, seizures, and even death, and are often referred to as “water intoxication” [4]. Up to 5% of chronic inpatients develop water intoxication, although mild cases may go undetected [4].

Neuroendocrine studies revealed that schizophrenia patients with hyponatremia secreted excess antidiuretic hormone (ADH) in a hypo-osmolemic serum state [5,6]. ADH controls the reabsorption of water molecules in the tubules of the kidneys and plays a key role in the regulation of water. Therefore, excessive ADH secretion may accelerate a hyponatremic state and produce water intoxication [6,7]. The pathophysiology of polydipsia on the other hand remains to be elucidated.

Previous imaging studies indicate the brains of polydipsic hyponatremic schizophrenia patients present with volume reductions in the anterior medial temporal lobe [8,9], especially in the anterior hippocampus relative to nonpolydipsic schizophrenia using region of interest (ROI) analysis [10]. Indeed, one study reported that polydipsic, hyponatremic schizophrenia patients exhibited bilateral inward deformations in the anterior lateral hippocampal surface [11]. However, structural differences in other brain regions of these polydipsic hyponatremic schizophrenia patients were not assessed. Interestingly, hyponatremic patients presented with poorer neuropsychological functioning compared to patients without a water imbalance [12-15]. Polydipsic hyponatremic schizophrenia patients scored lower on the Mini-Mental State Examination (MMSE) [14] with significant impairments in visual memory and information processing [12], intelligence, learning/memory, and facial discrimination [15] compared to control patients. Thus, it is considered that the structural changes in the anterior hippocampus, as well as in the associated prefrontal/limbic brain regions contribute to the underlying pathophysiology in polydipsic hyponatremic schizophrenia [11,15]. To our knowledge, these brain regions have not been examined using voxel-based morphometry (VBM) [16] in polydipsic schizophrenia patients. VBM is an efficient whole brain unbiased technique capable of analyzing structural magnetic resonance images (MRI), such as the differences between brains of schizophrenia patients with or without water imbalances. Lastly, previous studies [15] have not evaluated executive functions, which are impaired in schizophrenia [17-20], nor have they included healthy control subjects.

To assess the pathophysiology of polydipsic schizophrenia (PS), we used the VBM approach and evaluated neuropsychological function, including executive functions, to compare the difference between PS, nonpolydipsic schizophrenia (NS) and healthy controls (HC). In the VBM analysis, the main aim of this study was to compare brain structural volumes between PS and NS. As a preliminary exploration, we also compared PS with HC.

## Methods

### Subjects

Patients were recruited from among inpatient and outpatient facilities at the Nara Medical University Hospital and affiliated hospitals. The patients fulfilled the Diagnostic and Statistical Manual of Mental Disorders, 4th edition (DSM-IV) criteria for schizophrenia. Similar to a previous study [21], the diagnosis of polydipsia was made when the patient was observed to consistently drink excessive quantities of fluid by the hospital staff or the patients’ family. To ensure the validity of a diagnosis of polydipsia, we limited our study to only those patients with a history of polydipsic episodes and low serum sodium levels (less than 135 mEq/L) spanning at least the three most recent years per medical records. Therefore, all PS patients had a history of both excessive fluid intake and hyponatremia within three years. Research psychiatrists confirmed that these patients’ hyponatremia was associated with polydipsia and was not attributable to medical disorders such as hypothyroidism, or cardiac, renal, or hepatic failure, or to medications such as diuretics or carbamazepine. The latter, carbamazepine, can induce hyponatremia via the syndrome of inappropriate antidiuretic hormone secretion (SIADH) [22]. We defined PS as patients with a history of at least two hyponatremic states induced by polydipsia on available records, and who were continuously treated with water restrictions even on the day of testing because of their excessive fluid intake but who were not isolated from sources of water supply. In summary, PS was diagnosed based not only on a history of hyponatremia and excessive drinking fluid spanning the three most recent years but also on the patients’ current treatment situation (e.g., restriction of drinking excessive quantity of fluid). NS had no history of polydipsia or serum sodium levels lower than 135 mEq/L. One experienced psychiatrist assessed the severity of symptoms using Positive and Negative Syndrome Scale (PANSS) [23]. As presented in detail in Table 1, eight male right-handed PS (43.75 ± 6.58 years) and eight male right-handed NS (43.63 ± 7.67 years) were enrolled in this study. All of these patients were inpatients, except one PS and two NS. The frequency of hyponatremic states was 5.1 ± 2.9 times on available records, and the duration of polydipsia was 8.4 ± 4.7 years in PS group. For comparison, eight male right-handed HC (44.75 ± 3.77 years) patients were recruited from the community in Nara by word of mouth. Exclusion criteria for all participants were: any current neurological disorders, a family history of inheritable neurological disorders, and a history of head injury resulting in loss of consciousness, as well as alcohol or substance abuse. This study was approved by the Ethics Committee of the Nara Medical University. After a complete description of the study to each participant, written informed consent was obtained. To help rule out the possible effects of hydration status on whole brain volume [24] and neuropsychological functioning [15], plasma osmolalities were obtained on the day of testing. All participants were administered MRI and neuropsychological tests on the same day at Nara Medical University.

**Table 1:** Demographic and clinical characteristics

**mean ± S.D.**	**PS (n = 8)**	**NS (n = 8)**	**HC (n = 8)**	**Mann-Whitney U or Kruskal-Wallis**	**p-value**
Age [years]	43.75 ± 6.58	43.63 ± 7.67	44.75 ± 3.77	0.74	0.69
Body height [cm]	167.35 ± 5.24	170.04 ± 6.70	170.25 ± 4.65	1.76	0.414
Body weight [kg]	64.31 ± 7.94	70.48 ± 22.25	70.13 ± 8.36	2.4	0.302
Education [years]	13.38 ± 2.77	14.25 ± 1.58	14.38 ± 2.00	0.49	0.784
Posm [mmol/kg]	287.32 ± 11.82	289.57 ± 3.77	288.50 ± 4.04	0.64	0.727
Duration of illness [years]	20.38 ± 7.41	13.31 ± 9.63	-	17	0.114
No. of polydipsic episodes	5.1 ± 2.9	-	-		
Duration of polydipsia [years]	8.4 ± 4.7	-	-		
CPZ equi [mg/day]	859.38 ± 520.68	525.0 ± 287.85	-	21.5	0.269
PANSS Positive	17.5 ± 1.85	17.38 ± 3.20	-	33	0.913
PANSS Negative	26.13 ± 2.8	25.38 ± 1.41	-	30.5	0.871
PANSS General	48.63 ± 2.62	47.38 ± 2.62	-	23.5	0.365

*S*.*D*. standard deviation, *PS* polydipsic schizophrenia, *NS* nonpolydipsic schizophrenia, *HC* healthy controls, *Posm* plasma osmolality, *CPZequi* chlorpromazine equivalents, *PANSS* positive and negative syndrome scale.

### MRI acquisition and processing

Three-dimensional T1-weighted images were obtained on a 1.5 T MRI machine (Magnetom Sonata, Siemens AG, Erlangen, Germany) at Nara Medical University. All slices were sagittal slices across the entire brain with a repetition time = 1500 ms, inversion time = 800 ms, echo time = 3.93 ms, flip angle = 15°, field of view = 230 mm, slice thickness = 1.25 mm without gap, and a matrix size = 512 × 512.

Image analysis was performed using statistical parametric mapping (SPM) 5 software (http://www.fil.ion.ucl.ac.uk/spm/software/spm5/) developed in the Wellcome Department of Imaging Neuroscience, Institute of Neurology, University College London, running in MATLAB 7.8.0 (Mathworks, Sherborn, MA, USA).

VBM in SPM5 combines tissue segmentation, bias correction, and spatial normalization into a unified model [25] using default parameters. Individual brains were normalized to tissue probability maps provided by the International Consortium for Brain Mapping—East Asian brains. Segmented gray matter (GM), white matter (WM) and cerebrospinal fluid (CSF) images in native spaces were used to check the segmentation in that space and to measure total intracranial volume (TIV). Modulated normalized GM and WM images were tested for voxelwise differences in the relative volume of GM and WM. After segmentation, the GM and WM images were smoothed to a full-width half maximum Gaussian kernel of 8 mm.

### Neuropsychological test

The Brief Assessment of Cognition in Schizophrenia (BACS) [26] is an instrument that assesses multiple aspects of cognition. The domains of neuropsychological functions evaluated by the BACS are verbal memory, working memory, motor speed, verbal fluency, attention/speed of processing, and executive function. The Brief Assessment of Cognition in Schizophrenia, Japanese version (BACS-J) is Japanese version of BACS and has been tested and shown to have reliability and validity [27]. The time required for BACS-J testing is about half an hour. The same clinical psychologist administered the BACS-J and gauged the neuropsychological functions. The primary measure for each test in the BACS-J was standardized by creating z-scores and setting the HC mean to zero and the standard deviation to one. A composite score was calculated by averaging all of the six standardized primary measures from the BACS-J.

### Statistical analysis

The Statistical Package for the Social Science (SPSS) version 16.0 for Windows was used for statistical analysis of demographic characteristics, clinical characteristics and neuropsychological data. We used the Mann-Whitney *U* test and Kruskal-Wallis test to determine statistical significance. For multiple comparisons, post-hoc tests were applied using the Dunnett T3 test. A significance level at p = 0.05 was used.

VBM analysis using SPM5 was conducted for the group comparisons between PS vs. NS and PS vs. HC. Age and TIV were used as nuisance variables, and an absolute masking threshold of 0.2 was used. Output was in the form of SPM with the Montreal Neurological Institute (MNI) coordinates based on a height threshold of p < 0.001 uncorrected and an extend threshold of 50 contiguous voxels. Neuroanatomical locations were identified using the WFU Pick Atlas Tool (http://www.ansir.wfubmc.edu/).

## Results

### Demographic and clinical characteristics

The comparisons of the three groups with respect to demographic and clinical characteristics are presented in Table 1. There were no differences in demographic and clinical characteristics among the three groups. All groups had normal hydration status on the measuring day. PS had a longer duration of illness and higher chlorpromazine equivalent doses than did NS, but there were no significant differences between these two patient groups. Also, psychopathological scores measured by PANSS were not significantly different between these two patient groups.

### Brain structural differences

#### PS versus NS

**Figure 1:**
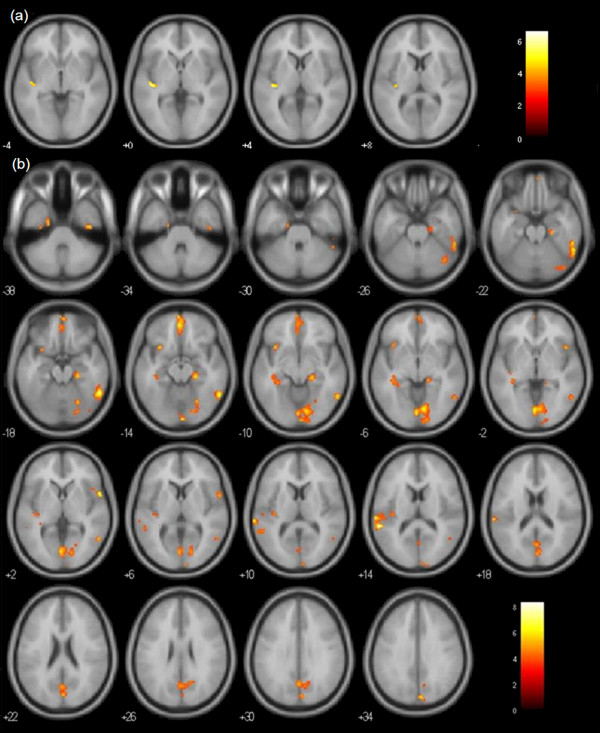
**Significant regional GM**/**WM volume reduction.** (**a**) PS < NS. (**b**) PS < HC.P < 0.001 uncorrected with an extent threshold of 50 contiguous voxels displayed on the MNI template (avg152T1). The right side of each section represents the right side of the brain. The *z* coordinate in MNI space is indicated in each section.

PS showed significantly reduced GM/WM volume in the left insula compared with NS (Figure 1a, Table 2). No significant increased regions were found in NS compared with PS.

**Table 2:** **Regional GM**/**WM volume changes**

**Between group comparison**	** Decreased region**	**MNI coordinates (mm)**			**voxels**	**t-value**
		**X**	**Y**	**Z**		
PS < NS	L insula	−42	−22	0	73	6.58
PS < HC	R inferior temporal gyrus	56	−52	−18	471	7.28
	L superior temporal gyrus	−58	−40	14	289	8.44
	R superior temporal gyrus	58	8	2	89	6.89
	L insula	−38	−22	12	73	5.09
	L medial frontal gyrus	−2	50	−14	315	6.39
	L inferior frontal gyrus	−30	16	−14	94	5.67
	R lingual gyrus	10	−78	−8	1055	6.93
	R cuneus	4	−82	32	420	6.08
	R parahippocampal gyrus	22	−28	−14	194	6.18
	L uncus	−22	−6	−38	75	5.20
	R cerebellar posterior lobe	40	−66	−26	92	5.01

*MNI* Montreal Neurological Institute, *PS* polydipsic schizophrenia, *NS* nonpolydipsic schizophrenia, *HC* healthy controls, *R* right, *L* left.

#### PS versus HC

PS showed widespread GM/WM volume reduction compared with HC (Figure 1b, Table 2). No significant increased regions were found in PS compared with HC.

### Neuropsychological differences

Neuropsychological scores are shown in Table 3. The composite score was −2.57 ± 1.78 for PS, –1.69 ± 1.69 for NS and 0.00 ± 0.95 for HC. PS group was significantly more impaired than HC in all categories of BACS-J. Furthermore, PS group was more impaired in all categories of BACS-J than NS, although not significantly. NS group was significantly more impaired than HC in motor speed, attention/speed of processing, and executive function. HC group did not show any impairment in all BACS-J subcategories relative to those patient groups.

**Table 3:** **Neuropsychological scores using the BACS**-**J**

	**PS (n = 8)**	**NS (n = 8)**	**HC (n = 8)**	**Kruskal-Wallis**	**p-value**	**post hoc**
verbal memory	21.0 ± 12.47	30.25 ± 8.31	38.13 ± 8.95	8.3	<0.05	PS < HC
working memory	15.38 ± 2.13	17.13 ± 5.62	22.38 ± 3.78	9.1	<0.05	PS < HC
motor speed	40.75 ± 13.18	52.75 ± 15.34	72.75 ± 9.68	14.4	<0.001	PS < HC, NS < HC
verbal fluency	29.75 ± 7.91	33.38 ± 13.64	42.38 ± 6.78	6.1	<0.05	PS < HC
attention/speed of processing	39.88 ± 9.72	46.75 ± 15.27	65.75 ± 10.32	12.8	<0.01	PS < HC, NS < HC
executive function	13.88 ± 3.80	15.50 ± 2.67	18.63 ± 1.19	10.2	<0.01	PS < HC, NS < HC

*PS* polydipsic schizophrenia, *NS* nonpolydipsic schizophrenia, *HC* healthy controls.

## Discussion

This is the first study to assess brain structural changes using the VBM approach and neuropsychological impairment—including executive function—between PS, NS, and HC. The current study demonstrated PS showed significant volume reduction in the left insula compared to NS. In imaging studies of schizophrenia, the laterality of the insula volume reduction is inconsistent—localization can be found on either the right side, left side, or bilaterally [28]. In our study, there were no significant differences in the clinical characteristics between the two patient groups. In particular, the PANSS scores of PS and NS were almost similar, despite previous findings indicating that schizophrenia with polydipsia were characterized by greater severity of illness [29]. Therefore, the left-sided volume reduction seemed to be attributable to either the polydipsia or the secondary hyponatremia in our study. The insula is a component of the “limbic integration cortex” and is associated with emotional and neuropsychological functions [30-32]. Although there were no significant differences in neuropsychological scores between the patient groups, the PS group was more impaired in all categories of the BACS-J compared to the NS group. This may be due to their reduced insula volume, which in turn may indirectly affect neuropsychological function. Indeed, the insula has extensive connections to many brain areas including limbic regions [28]. It should be noted that chronic hyponatremia may also be associated with psychomotor and attention deficits [33,34]. Although in the current study the PS group had normal hydration status on the day of testing, it is possible that past or present hyponatremia affects neuropsychological function.

In a preliminary exploration, we found that PS had widespread GM/WM volume reduction and neuropsychological impairment compared to HC. It is well accepted that fronto-temporal integration [35], prefrontal-parietal networks [36], the fronto-parieto-occipital network [37] and the cortical-thalamic-cerebellar-cortical circuit [38] support a variety of neuropsychological functions. The widespread GM/WM volume reduction of these brain networks, as in PS, would result in neuropsychological impairment. Previous research has indicated that polydipsic hyponatremic patients had reduced anterior hippocampi compared to nonpolydipsic patients [10] and that reduced anterior hippocampi were associated with chronic hyponatremia [15]. The uncus is regarded as the anterior region of the hippocampus [39] and is a recognizable internal marker for dividing the hippocampus [40,41]. Although in this study the size of the uncus was not significantly different between the two patient groups, PS exhibited volume reductions in the left uncus compared with HC. We recruited PS patients who were treated with water restriction. These water restrictions may act to decrease the hyponatremic status frequency and may prevent further volume reduction of the anterior hippocampus. In our study, PS showed normal hydration status on the day of testing and normal sized brain regions when compared to the other groups. It has been previously reported that acute hyponatremia induced by polydipsia results in cerebral edema [24]. Therefore, our findings could reflect the trait abnormalities of PS.

There are some limitations in the current study. The first is the small sample size, which reflects the difficulty of recruiting and studying polydipsic patients [10,15]. It has been shown that longer duration of illness [42] and greater intensity of antipsychotic treatment [43] are probably associated with structural brain changes. Although the duration of illness and the chlorpromazine equivalent doses were not statistically different between PS and NS, their variances were moderate given our sample size. Therefore, we could not completely exclude the possibility of these confounding effects. The second limitation is that the participants in our study consisted only of males. It should be noted that a previous study found that polydipsic schizophrenics had a more frequent history of alcohol abuse [29], which was one of the exclusion criteria in our study. Thus, these factors might limit the generalizability of our results. Furthermore, our PS patients had histories of hyponatremia, but did not indicate continuous hyponatremia or were not examined with frequent hydration state measurements. Therefore, we could not identify if the hyponatremia or the polydipsia had more influence on the structural brain changes. To clarify this point, another study is needed that assesses polydipsic patients without a history of hyponatremia.

## Conclusion

Finally, it is possible that polydipsia or the secondary hyponatremia might induce a left insula volume reduction. Our findings provide insights on the pathophysiology of schizophrenic polydipsia. In addition, further insula volume reductions might induce further neuropsychological impairments. In the future, another imaging method like diffusion tensor imaging or functional imaging may help elucidate the brain networks disrupted or the extent of pathophysiology in polydipsia.

## Competing interests

The authors declare that they have no competing interests.

## Authors’ contributions

TN, MI and TK designed the study, wrote the protocol, and wrote the first draft of the manuscript. SK, KK, JK, KO, NK, TT and KK undertook the data management and performed the statistical analysis with TN. All authors contributed to and have approved the final manuscript.

## Pre-publication history

The pre-publication history for this paper can be accessed here:

http://www.biomedcentral.com/1471-244X/12/210/prepub
